# OTUB2 regulates KRT80 stability via deubiquitination and promotes tumour proliferation in gastric cancer

**DOI:** 10.1038/s41420-022-00839-3

**Published:** 2022-02-02

**Authors:** Siwen Ouyang, Ziyang Zeng, Zhen Liu, Zimu Zhang, Juan Sun, Xianze Wang, Mingwei Ma, Xin Ye, Jianchun Yu, Weiming Kang

**Affiliations:** 1 Chinese Academy of Medical Sciences and Peking Union Medical College, Beijing, China; 2 Department of General Surgery, Peking Union Medical College Hospital, Beijing, China

**Keywords:** Gastric cancer, Ubiquitylation

## Abstract

OTUB2 is a deubiquitinating enzyme that contributes to tumor progression. However, the expression of OTUB2 and its prognostic importance in gastric cancer remain unclear. The expression of OTUB2 and KRT80 in GC tissues was investigated using western blotting, qRT-PCR, multiple immunofluorescence staining, and immunohistochemistry. For survival studies, Kaplan–Meier analysis with the log-rank test was used. The role of OTUB2 during GC proliferation was investigated using in vivo and in vitro assays. OTUB2 was found to be overexpressed in GC tissues and to act as an oncogene, which was linked to patients’ poor prognosis. Knockdown of OTUB2 inhibited the proliferative capacity of GC cells in vitro and in vivo, although the proliferative capacity was restored upon re-supplementation with KRT80. OTUB2 mechanically stabilized KRT80 by deubiquitinating and shielding it from proteasome-mediated degradation through Lys-48 and Lys-63. Furthermore, by activating the Akt signaling pathway, OTUB2 and KRT80 facilitated GC proliferation. In summary, OTUB2 regulates KRT80 stability via deubiquitination promoting proliferation in GC via activation of the Akt signaling pathway, implying that OTUB2 could be a novel prognostic marker.

## Background

Gastric cancer (GC) is one of the most common malignant tumors of the gastrointestinal [1, 2]. Globally, GC is the fifth most frequently diagnosed form of cancer and the fourth leading cause of death from cancer [3]. Over the last few decades, advancements in therapeutic and diagnostic methods have resulted in improvements in the prognosis of GC. However, in China, the prevalence of stage II-III GCs is as high as 58·0% [4]. While surgery is the primary treatment for GC, the long-term survival rate of patients with locally advanced gastric cancer (LAGC) following surgery is still less than 20–30% [5]. One of the primary reasons for poor prognosis is a dearth of effective biomarkers for prognosis prediction and treatment guidance. As a result, the identification of novel progression-linked biomarkers and elucidation of the underlying mechanisms are needed to enhance the prognosis of patients with GC.

Akt is a serine/threonine-specific protein kinase that is needed for the intracellular signaling of numerous growth factors, such as insulin. Due to the fact that the Akt pathway promotes cell survival and proliferation in living organisms, it is overexpressed in cancer cells [6–8]. Ubiquitination has been shown to be able to control the expression of this pathway. In most cases, when deubiquitinating enzymes (DUBs) are overexpressed, Akt expression increases, resulting in the growth of tumors [9]. Accordingly, identifying oncogenes or suppressors of the Akt pathway and developing innovative drugs may be the most effective approach for managing LAGC and enhancing the prognosis of advanced-stage GC patients.

Posttranslational modifications to proteins are critical in regulating their function, interactions, subcellular localization, and stability. The ubiquitin-proteasome system (UPS) is the main mechanism for posttranslational modification of proteins. It is regulated by E1, E2, and E3 ubiquitin ligases and DUBs. The ubiquitin-mediated degradation of proteins is a reversible mechanism that involves the sequential action of ubiquitin-activating enzymes E1, ubiquitin-conjugating enzymes E2, and ubiquitin ligases E3. It catalyzes the ubiquitination of substrates, thus directing their degradation to the proteasome. DUBs catalyze the ubiquitin elimination from polyubiquitin chains [10–12]. According to current research, the human genome encodes approximately 100 DUBs, of which the ovarian tumor-like proteases (OTU) family is a member. The OTU family is highly specific for identifying distinct ubiquitin chain types, and it can be further subdivided into the OTUB1 and OTUB2 [13–15]. OTUB2 inhibits RAP80’s K63 ubiquitin chain synthesis, thus impairing non-homologous recombination repair during the early stages of DNA damage repair [16]. Additionally, OTUB2 plays a pivotal role in the tumorigenesis of non-small cell lung cancer via the stabilization of U2AF2 and activation of the AKT/mTOR pathway [17]. However, there are relatively few studies on OTUB2, and our understanding of its role in tumor progression is limited, especially in GC.

Keratin 80 (KRT80), an epithelial keratin, has been described as a factor in endocrine-resistant ER breast cancer that promotes cytoskeletal changes and invasive conduct [18]. And KRT80 was expected to communicate with PRKDC in order to facilitate the migration and invasion of colorectal cancer cells by activating the Akt pathway [19]. Additionally, via the PI3K/Akt pathway, the circPIP5K1A/miR-671-5p/KRT80 axis facilitates GC progression [20].

In our study, we discovered that OTUB2 and KRT80 were both upregulated concurrently in GC tissues and were linked with a bleak patient prognosis. This is the first time, we demonstrate that OTUB2 functions as a brand-new catalyst of GC by deubiquitinating KRT80. Besides that, OTUB2 activates the Akt pathway, suggesting that it may be a novel therapeutic and prognostic target in GC.

## Results

### OTUB2 and KRT80 are simultaneously overexpressed in GC and are linked with poor prognosis

**Fig. 1:**
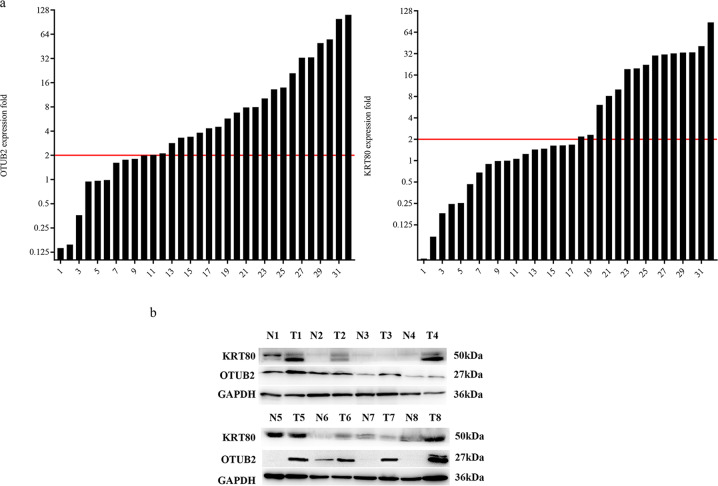
OTUB2 and KRT80 are expressed in gastric cancer tissues. **a** Real-time PCR analysis of the expression of OTUB2 and KRT80 in 32 primary gastric cancer tissues and paired normal tissues. The levels of mRNA expression were normalized to those of β-actin. **b** Western blotting was used to determine the expression levels of OTUB2 and KRT80 proteins in eight representative gastric cancer specimens; “N” denotes adjacent normal tissue and “T” denotes tumor tissue. At least three separate experiments are represented by the data.

To evaluate whether the expression of OTUB2 and KRT80 in GC are consistent with our previous research on public databases, we analysed mRNA expression levels in GC and paired adjacent tissues obtained randomly from our pathology center using qPCR. The results indicated that 68.75% (22/32) of GC tissues manifested higher OTUB2 mRNA expression compared with the corresponding adjacent normal tissues, however, 46·88% (15/32) of GC tissues showed higher KRT80 mRNA levels (Fig. 1a). It seems that the mRNA levels of OTUB2 and KRT80 are somewhat inconsistent. Then we examined the protein levels. The western blotting analysis of eight randomly selected pairs showed that both OTUB2 and KRT80 protein expression were simultaneously (6/8) upregulated in GC tissues compared with paired adjacent normal tissues (Fig. 1b). These results indicated that the upregulation in the protein levels of KRT80 may be due to some posttranscriptional reasons.

**Fig. 2:**
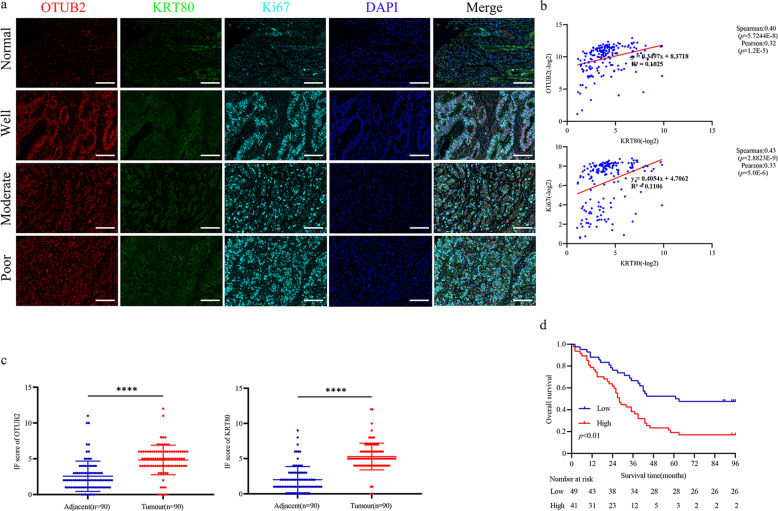
Multiple immunofluorescence (IF) staining analysis in a tissue microarray (TMA). **a** Representative multiple fluorophore-antibody (red: OTUB2; green: KRT80; cyan: Ki67; blue: DAPI) staining on the TMA. Scale bar, 400 μm. **b** Correlation analysis of KRT80 with OTUB2 and KRT80 with Ki67 protein expression on TMA, respectively, *P* < 0.05 indicates a significant outcome. **c** IF staining scores of OTUB2 and KRT80 expression in 90 paired GC samples. **d** Kaplan–Meier analysis with a log-rank test was performed in GC patients with varying levels of OTUB2 protein expression. ****P < 0.0001, Student’s *t*-test.

To investigate further the correlation between OTUB2 and KRT80 protein expression levels in GC. Multiple immunofluorescence (IF) staining analysis was performed to evaluate OTUB2 and KRT80 expression in a tissue microarray (TMA) consisting of 90 cases of primary gastric cancer with different tumor differentiation and paired adjacent normal tissues (Fig. 2a). We analysed the correlation between OTUB2 and KRT80 by the positive rate of staining. The results manifested OTUB2 and KRT80 were positively correlated in GC and paired adjacent normal tissues. We also analysed the correlation between KRT80 and Ki67, which is the most frequently used proliferation marker in IF and immunohistochemistry (IHC) [21]. The results showed KRT80 and Ki67 were similarly positively correlated in coincidence with OTUB2 and KRT80 (Fig. 2b).

Among the 90 paired tissues samples in the TMA, the protein expression levels of OTUB2 and KRT80 were upregulated in GC compared with paired adjacent gastric tissues (Fig. 2c). We further analysed correlations between OTUB2 protein expression level and GC clinicopathological parameters. 90 GC patients were split into OTUB2 low-expression (54.44%, 49/90) and high-expression (45.56%, 41/90) groups in accordance with IF staining. We found that high expression levels of OTUB2 were positively correlated with tumor T stage (*P* = 0.001), American Joint Committee on Cancer (AJCC) stage (*P* = 0.006), and differentiation (*P* = 0.010), while there was no significant correlation between OTUB2 protein level and age, gender, tumor location, tumor N stage, or tumor M stage (*P* > 0.05) (Table 1). Moreover, Kaplan–Meier survival analysis with log-rank testing was used to determine the prognostic role of OTUB2 in predicting survival in GC patients. It was discovered that patients with high OTUB2 protein expression had a lower overall survival (OS) rate than those with low OTUB2 protein expression (*P* < 0.01) (Fig. 2d). Taken together, both OTUB2 and KRT80 proteins are overexpressed in GC simultaneously. And the high expression of OTUB2 correlates with the poor prognosis of GC patients. All these data manifested OTUB2 and KRT80 upregulation may be associated with the tumorigenesis and proliferation of GC.

**Table 1:** Association between clinicopathological parameters and OTUB2 expression (*n* = 90).

	OTUB2 expression		*P* value
	Low (*n* = 49)	High (*n* = 41)	
Gender			
Male	37	33	0.572
Female	12	8	
Age, years			
<60	15	14	0.721
≥60	34	27	
Tumor location			
Fundus	6	8	0.683
Antrum	27	20	
Lesser curvature	13	9	
Greater curvature	3	4	
Tumor T stage			
1	4	0	0.001*
2	18	9	
3	14	27	
4	13	5	
Tumor N stage			
0	14	9	0.276
1	8	8	
2	10	15	
3	17	9	
Tumor M stage			
0	48	38	0.226
1	1	3	
AJCC stage			
I	5	2	0.006*
II	23	7	
III	20	29	
IV	1	3	
Differentiation			
Well	16	6	0.010*
Moderate	30	24	
Poor	3	11	

*P* value is based on Pearson’s chi-square test.

*P < 0.05 indicates a significant association among the variables.

### OTUB2-promoting gastric cancer cells proliferation is coincident with KRT80 in vitro

**Fig. 3:**
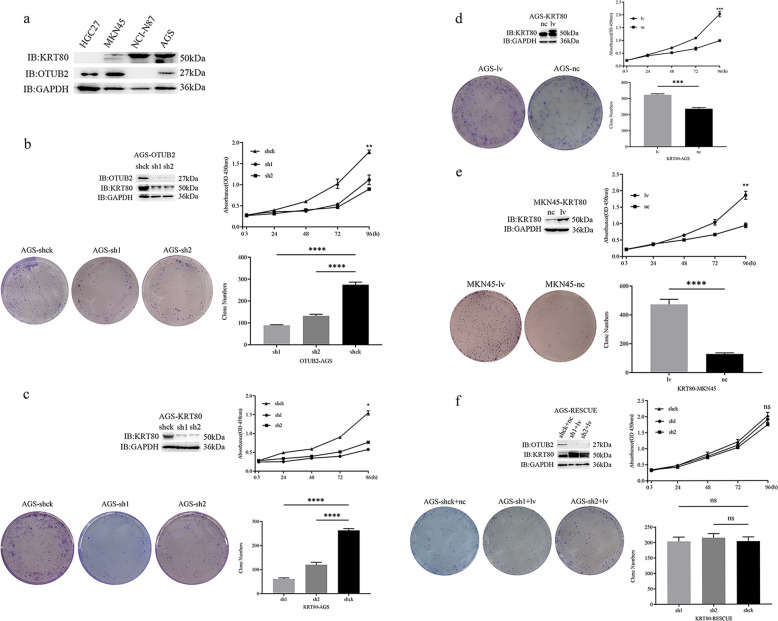
OTUB2-promoting gastric cancer cell proliferation is coincident with KRT80 in vitro. **a** Western blotting analysis of OTUB2 and KRT80 expression in four gastric cancer cell lines. **b**–**f** Western blot analysis, CCK-8 assay, plate colony formation assay, and quantification of OTUB2 and KRT80 expression and cellular growth in AGS cells and MKN45 cells with stable OTUB2-knockdown, KRT80-knockdown, KRT80-overexpression, KRT80-overexpression, and rescued-KRT80 respectively. The number of gastric cancer cell clones was quantified. Error bars represent the mean ± SD of three independent experiments. *P < 0.05; **P < 0.01; ***P < 0.001; ****P < 0.0001; ns not significant (Student’s *t*-test).

To investigate the function of OTUB2 and KRT80 in gastric cancer cells, we conducted a western blotting analysis of four gastric cancer cell lines first (Fig. 3a). We found that AGS cells show high KRT80 and OTUB2 expression and that MKN45 cells show low KRT80 expression, although the expression of OTUB2 and KRT80 was not so consistent in the four cell lines. Then we developed OTUB2-knockdown AGS and KRT80-knockdown AGS cells and KRT80-overexpression MKN45 to investigate OTUB2 and KRT80 in gastric cancer with the aid of lentivirus technology. OTUB2-knockdown efficiency through western blotting and cell development through CCK-8 proliferation assays and platform colonization tests have shown that the OTUB2-knockdown reduced proliferative capacities of AGS cells significantly when compared with negative control cells (Fig. 3b). Interestingly, the KRT80-knockdown results in AGS cells showed that the limited proliferative capacity was consistent with OTUB2 (Fig. 3c). Meanwhile, KRT80-overexpression efficiency and cellular growth were evaluated in different cell lines (Fig. 3d, e). The results showed that overexpressing KRT80 dramatically promotes the cell growth capacity compared with the control group in MKN45 cells, which is coincident with AGS cells. We also tested the OTUB2-knockdown AGS mRNA level. Intriguingly, KRT80’s mRNA level was not substantially different from a knockdown to control groups (Fig. S1), although KRT80’s protein level has been drastically affected as OTUB2 is knocked down (Fig. 3b). This may also mean that the compromise of KRT80 protein is caused by posttranscriptional factors and is linked to OTUB2. To investigate further whether knockdown of OTUB2 specifically regulates the expression of the KRT80 protein, we have rescued KRT80 from AGS cells that previously knocked OTUB2 by lentiviral methods. As shown in (Fig. 3f), the growth and proliferation abilities of AGS-RESCUE cells were no different from the control group in a statistically significant way. These data indicated that the growth and proliferation capacity originally impaired in OTUB2-knockdown AGS cells was restored by replenishment of KRT80. Taken together, both OTUB2 and KRT80 positively promote growth and proliferation in gastric cancer cells in vitro, and OTUB2 also specifically regulates the expression of KRT80 proteins.

### OTUB2 interacts with and stabilizes KRT80

**Fig. 4:**
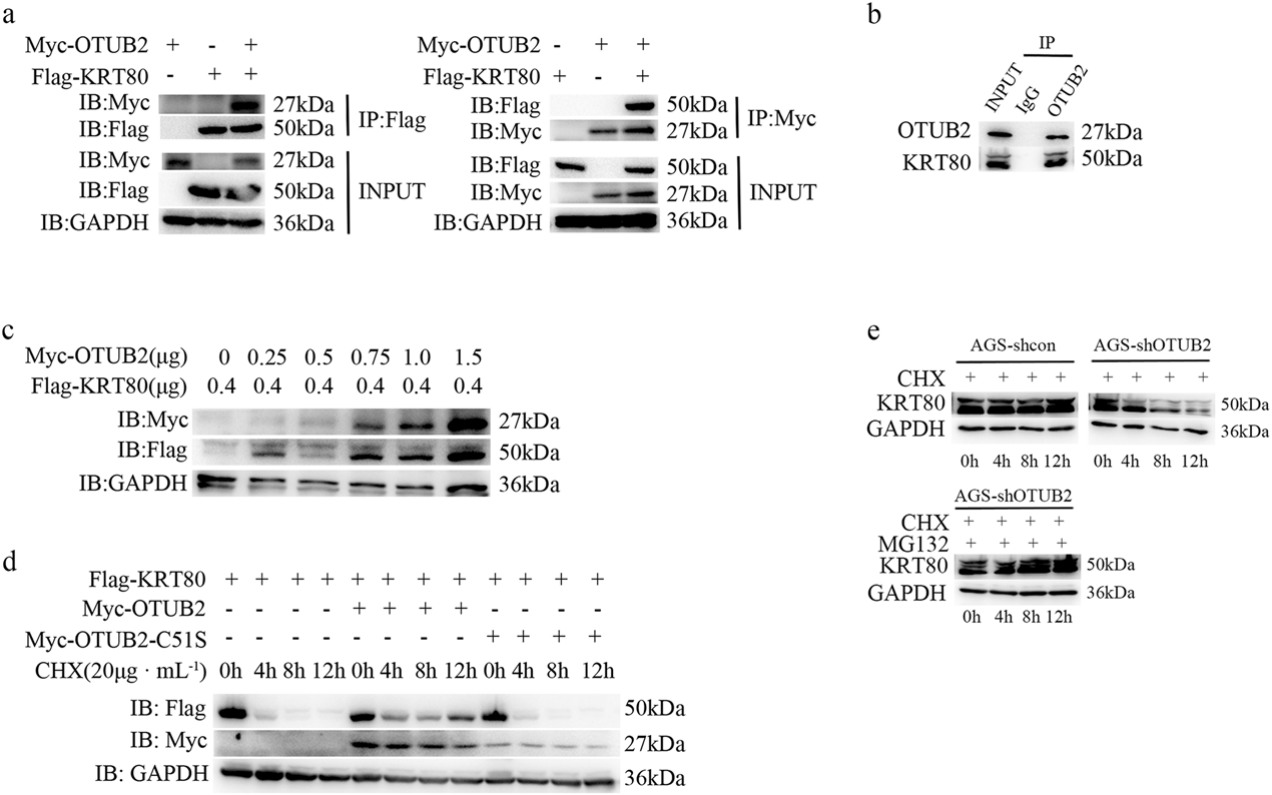
OTUB2 interacts with and stabilizes KRT80. **a** Reciprocal co-IP of OTUB2 and KRT80. Plasmids encoding Myc-tagged OTUB2 and Flag-tagged KRT80 were co-transfected into HEK293T cells. Cell lysates were immunoprecipitated with anti-Flag or anti-Myc antibodies, followed by western blotting as indicated. **b** Endogenous co-IP of OTUB2 and KRT80 in AGS cells. Anti-OTUB2 and anti-KRT80 antibodies were used to immunoprecipitate cell lysates, which were then identified by western blotting. **c** Flag-tagged KRT80 (0.4 μg) was co-transfected into HEK293T cells with the specified doses of Myc-OTUB2 (0.25, 0.5, 0.75, 1, and 1.5 μg) and Myc vector. After 48 h, the transfected cells were harvested and western blotting analysis was performed. **d** Effects of OTUB2 on the degradation of KRT80 in HEK293T cells. Flag-tagged KRT80 was co-transfected into HEK293T cells with or without Myc-tagged OTUB2 or OTUB2-C51S and treated with CHX at various time points (0, 4, 8, and 12 h) prior to harvesting. The amount of Flag-tagged KRT80 protein was determined using an anti-Flag antibody. **e** Effects of OTUB2 on the degradation of KRT80 in gastric cancer cells. AGS-shck/shOTUB2 cells were exposed to CHX (20 μg/mL) and harvested at the indicated times. KRT80 was measured using western blotting. KRT80 was stabilized when cells were co-treated with CHX and MG132 (20 μM).

We were eager to understand the relationship between OTUB2 and KRT80. As we expected, we discovered an interaction between OTUB2 and KRT80. As shown in (Fig. 4a, b), exogenous OTUB2 coimmunoprecipitated with KRT80 in HEK293T cells and endogenous OTUB2 coimmunoprecipitated with KRT80 in AGS cells. We transfected a rising gradient of OTUB2 and an equivalent amount of KRT80 to further investigate the relationship between OTUB2 and KRT80. KRT80 was found to be more strongly expressed when OTUB2 was increased (Fig. 4c). The findings revealed that OTUB2 can dose-dependently stabilize KRT80. The protein synthesis inhibitor cycloheximide (CHX, 20 μg/mL) was used to the transfected HEK293T cells for the specified times to further confirm this conclusion, and the KRT80 protein expression was measured. Consistently, we found that in the presence of OTUB2, the half-life of the KRT80 protein was substantially extended, but not in the presence of the inactive-enzyme mutant OTUB2-C51S (Fig. 4d). Since OTUB2 primarily affects protein expression via ubiquitination, we gave GC cells CHX and the proteasome inhibitor MG132 (20 μM) respectively. We found that when compared to controls, knocking down OTUB2 resulted in lower protein stability of KRT80 when added CHX, however, the KRT80 degradation triggered by low OTUB2 expression was rescued when added MG132 (Fig. 4e). Collectively, these findings indicate that OTUB2 interacts with and stabilizes KRT80 and that it is likely to prevent KRT80 degradation by deubiquitination.

### OTUB2 deubiquitinates KRT80 through Lys-48-linked and Lys-63-linked deubiquitination

**Fig. 5:**
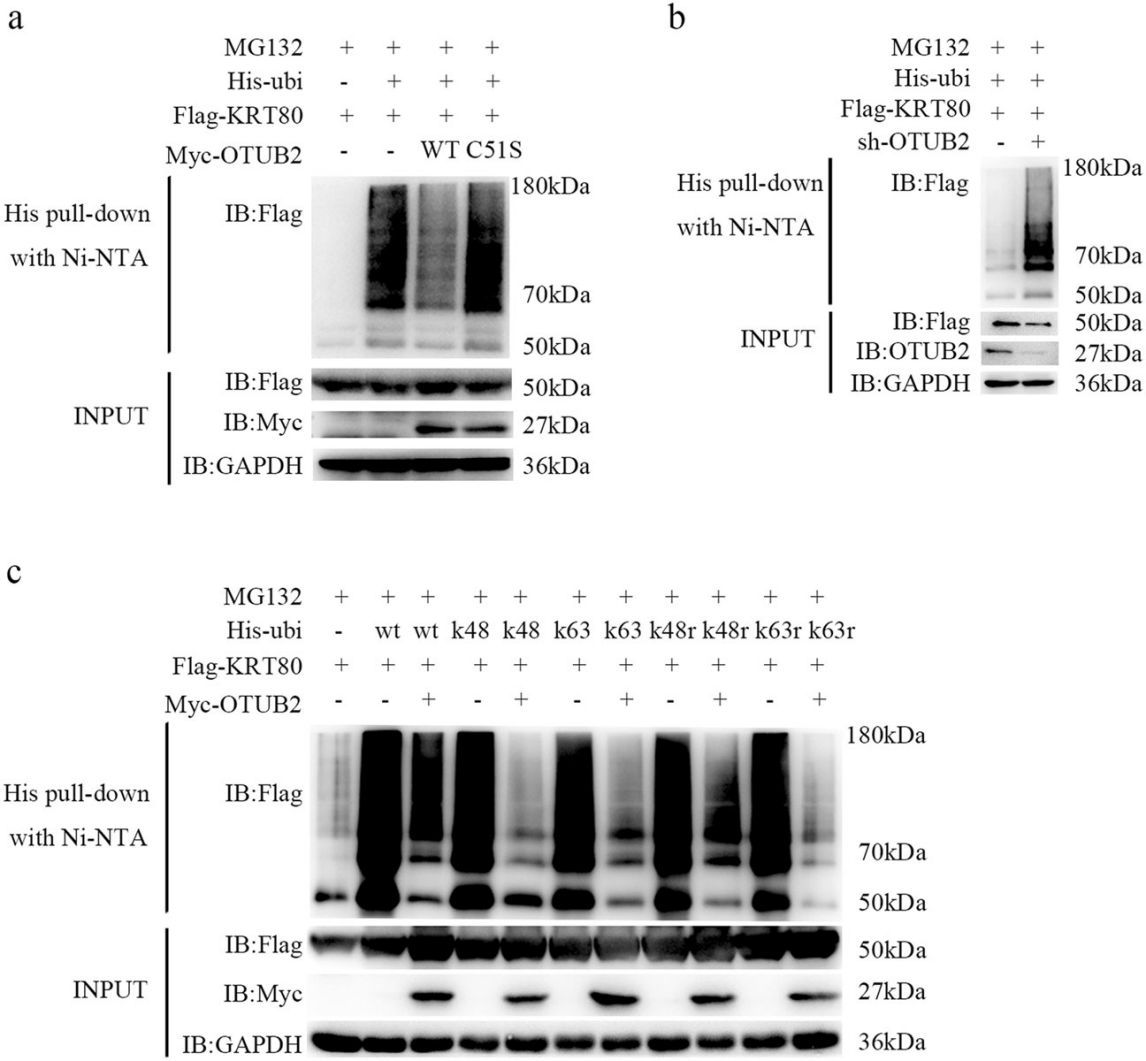
OTUB2 stabilizes KRT80 through Lys-48-linked and Lys-63-linked deubiquitination. **a** HEK293T cells were co-transfected with Flag-tagged KRT80, Myc-tagged OTUB2 (WT), Myc-tagged OTUB2-C51S (C51S), and His-ubiquitin, and then treated for 4 h with 20 μM MG132. As stated in the materials and methods section, the ubiquitin pull-down assay for Flag-tagged KRT80 was performed. **b** Roles of OTUB2 on KRT80 ubiquitination in AGS cells. Western blotting analysis showed polyubiquitination of KRT80. **c** Flag-tagged KRT80, Myc-tagged OTUB2 were co-transfected into HEK293T cells with His-ubiquitin and its mutants Lys-48 only, Lys-63 only, Lys-48r, and Lys-63r. Cell lysates were collected and ubiquitinated. KRT80 was observed using western blotting after being pulled down with nickel-nitrilotriacetic acid beads. The data reflect a minimum of three independent experiments.

With respect to the ubiquitin molecule, in general, is mainly made up of seven lysine residues (Lys-63, Lys-48, Lys-33, Lys-29, Lys-27, Lys-11, and Lys-6), and it is understood that each ubiquitin monomer can perform a different role by forming different polyubiquitin chains. Previous evidence has shown the functions of the Lys-48 and Lys-63 association ubiquitination primarily [22]. Polyubiquitin chains with a Lys-48 linkage are involved in the proteasome-dependent degradation of modified proteins, while polyubiquitin chains with a Lys-63 linkage are involved in DNA repair, signal transduction, and protein activity regulation [23]. To determine whether OTUB2 regulates KRT80 deubiquitination as previously hypothesized, co-transfection of KRT80-expressing plasmids into HEK293T cells with plasmids expressing either wild-type OTUB2 or its enzymatically inactive mutant OTUB2-C51S was performed. As predicted, the results indicated that OTUB2 was capable of significantly promoting KRT80 deubiquitination (Fig. 5a). In contrast, knocking down OTUB2 with shRNA increased the polyubiquitination of the KRT80 protein in AGS cells obviously (Fig. 5b). To further determine the form of polyubiquitin chain in KRT80 is removed by OTUB2, we co-transfected KRT80 with the Lys-63 only (K63) or Lys-48 only (K48) or Lys-63r (rest of Lys-48, K63r) or Lys-48r (rest of Lys-48, K48r) ubiquitin mutants and performed a His pull-down assay. We found that both K48 and K63 mutants are specifically involved in the OTUB2-mediated deubiquitination of KRT80 (Fig. 5c). In short, these results demonstrate that OTUB2 stabilizes KRT80 through both Lys-48-linked and Lys-63-linked deubiquitination, thereby preventing ubiquitin-mediated proteasome-dependent degradation of KRT80.

### OTUB2 expression is positively associated with the activation of the Akt signaling pathway

**Fig. 6:**
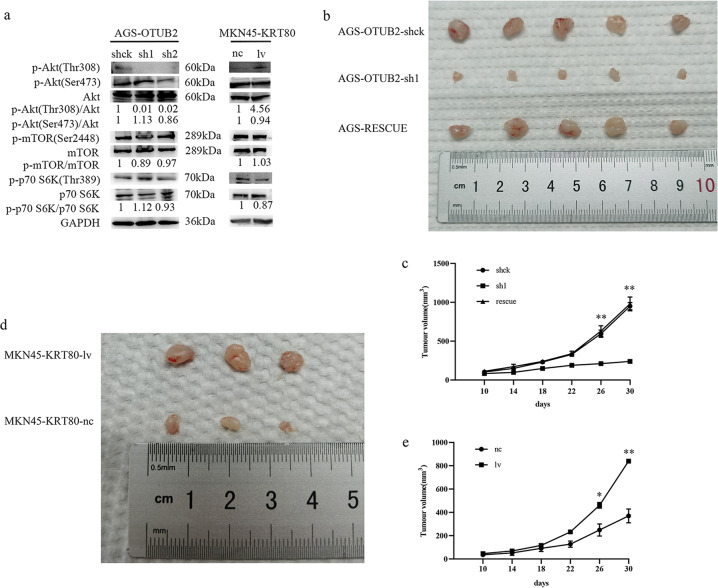
OTUB2 regulates KRT80, thereby activating the Akt signaling pathway, which results in the tumorigenesis and proliferation of GC in vivo. **a** Reduced Akt phosphorylation levels were detected by western blotting in OTUB2-knockdown AGS cells. Total Akt, mTOR, p70 S6K, and GAPDH served as controls. Increased Akt phosphorylation levels, as detected by western blotting, in KRT80-overexpressing MKN45 cells. **b**–**e** Effect of OTUB2-knockdown, KRT80 rescued and control AGS cells (**b**) or KRT80-overexpression MKN45 cells (**d**) on GC tumorigenesis in vivo. The volume of subcutaneous tumors was measured (*n* = 5 or *n* = 3). *P < 0.05; **P < 0.01 (Student’s *t*-test).

Several previous studies have shown that the phosphatidylinositol 3-kinase (PI3K)/Akt/mammalian target of rapamycin (mTOR)/70-kDa ribosomal protein S6 kinase (p70 S6K) pathway plays a critical role in regulating the growth of both normal and cancer cells [24, 25]. Via the PI3K/Akt pathway, the circPIP5K1A/miR-671-5p/KRT80 axis helps GC progression, according to a study of gastric cancer linked to KRT80 [20]. To determine whether OTUB2, like KRT80, regulates Akt pathway activation during the progression of GC, we analysed the expression levels of main Akt pathway proteins. Western blotting analysis showed that knockdown of OTUB2 in AGS cells downregulated phosphorylated Akt (p-Akt, Thr308) protein levels but did not have an impact on total Akt levels. Meanwhile, overexpression of KRT80 increased the amount of p-Akt (Thr308) protein in MKN45 cells. However, there were no notable changes in p-Akt (Ser473), phosphorylated mTOR (p-mTOR and Ser2448), mTOR, phosphorylated p70 S6K (p-p70 S6K and Thr389), and p70 S6K protein levels (Fig. 6a). These findings suggest that OTUB2 has the ability to directly activate the Akt signaling pathway, which is consistent with KRT80.

### OTUB2 regulates KRT80 to enhance tumorigenesis and proliferation of GC in vivo

**Fig. 7:**
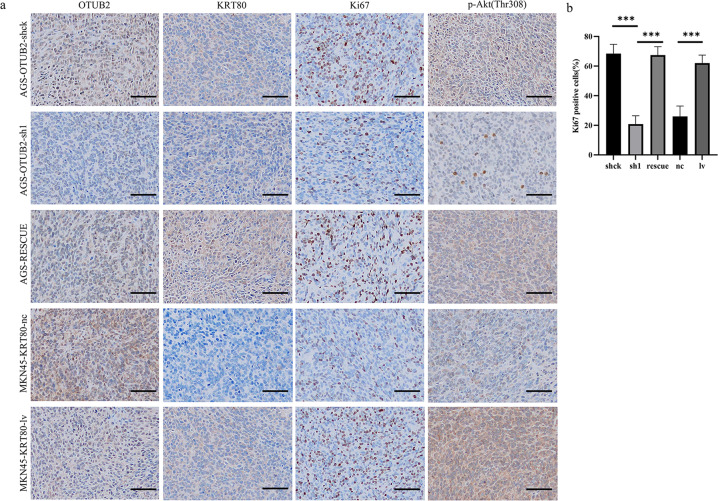
IHC staining in nude mouse xenograft tumors. **a** IHC staining of OTUB2, KRT80, Ki67, and p-Akt (Thr308) in nude mouse xenograft tumors derived from AGS-OTUB2-shck cells, AGS-OTUB2-sh1 cells, AGS-RESCUE cells, MKN45-KRT80-nc cells, and MKN45-KRT80-lv cells; scale bar 400 μm. **b** Ki67 positive cells quantitative analysis of IHC staining. ***P < 0.001 (Student’s *t*- test).

We constructed a subcutaneous xenograft model to examine the effects of OTUB2 and KRT80 on the proliferation and tumorigenesis of gastric cancer cells in vivo. Each group consisted of five mice. Two mice in the MKN45 group died because they did not adapt to the environment, so only three mice were left. As shown in (Fig. 6b, c), the volume of tumors produced by AGS cells transfected with OTUB2-sh1 plasmids was significantly less than that generated by controls, but the volume generated by AGS-RESCUE cells was recovered. Additionally, the average volume of tumor-derived from KRT80-overexpressing MKN45 cells grew faster than the control tumor volume (Fig. 6d, e). The results indicate that OTUB2 promotes the growth and proliferation of gastric cancer cells in vivo by specifically deubiquitinating the stability of KRT80. Moreover, when OTUB2 expression was decreased, KRT80 protein expression was decreased, inhibiting gastric cancer cells' growth capacity. Once KRT80 protein expression was restored, gastric cancer cells' growth ability was restored concurrently. IHC staining revealed that OTUB2-knockdown or KRT80-overexpression impaired or enhanced Ki67 protein expression in xenograft tumors, respectively, relative to controls. And Ki67 protein in AGS-RESCUE cells was also restored compared with AGS-OTUB2-sh1 cells (Fig. 7a, b) Notably, the expression of p-Akt (Thr308) protein coincided with the expression of KRT80 protein and Ki67 protein, suggesting that OTUB2 activates the PI3K/Akt signaling pathway by deubiquitinating KRT80, thus promoting the growth and proliferation of gastric cancer cells. In summary, these data confirm that KRT80 is indispensable for the proliferation of GC mediated by OTUB2.

## Discussion

GC progression is a complex process involving numerous molecular interactions that are controlled by key genes. Investigations into the regulatory mechanisms underlying these key genes may result in the creation of novel therapeutic strategies for GC patients. OTUB2, a member of the ovarian tumor-like proteases family, has been implicated in non-small cell lung cancer tumorigenesis and prognosis [17]. The deubiquitinating enzyme OTUB1 is known to participate in the classical RNF8 pathway for DNA damage repair [26] and is associated with esophageal cancer [27], bladder cancer [28]. OTUB2, on the other hand, is a much less well-known entity. We hypothesized that OTUB2 may have some previously unknown functions based on these findings. In this research, we discovered that OTUB2 expression was upregulated in gastric cancer and was significantly associated with the poor prognosis of patients. This is consistent with KRT80 protein expression in gastric cancer, but not with KRT80 mRNA expression. Given OTUB2’s position as a deubiquitinating protein, it was presumed that it correlated with KRT80 closely. Multiple immunofluorescence staining revealed a strong association between them. We then conducted cell proliferation experiments in vitro and discovered that both OTUB2 and KRT80 can promote the proliferation of gastric cancer cells.

However, OTUB2 and KRT80 were not so consistently expressed in the four cell lines. We believe that the progression of GC cell lines may not only be due to the role of OTUB2 and KRT80, but also other factors. That is why it appears that the near absence of OTUB2 expression in NCI-N87 and KRT80 expression in HGC27 do not affect the progression of these two cell lines. And the expression of OTUB2 and KRT80 proteins was not 100% consistent in gastric cancer and paired adjacent normal tissues (Fig. 1b). This is the focus of our future research, after all, cancer growth and proliferation are not so simple as a result of single or double factors.

We then investigated the relationship between the two and discovered that OTUB2 was indeed capable of stabilizing KRT80 by specifically deubiquitinating it via Lys-48 and Lys-63, thereby activating the Akt pathway and thus promoting gastric cancer cell proliferation. Perhaps OTUB2 can activate the Akt pathway on its own without requiring KRT80? The IHC results in (Fig. 6a, b) indicate that the response is negative. MKN45 cells express a high level of OTUB2 but very little Ki67 and p-Akt (Thr308), indicating that OTUB2 does not activate the Akt pathway on its own, but via KRT80. Those findings indicate that developing a molecular inhibitor of OTUB2 in the future may have a significant impact on the treatment of patients with LAGC.

Nevertheless, we have not yet discussed the association between OTUB2 and gastric cancer metastasis. Sumoylated OTUB2 interacts specifically with yet-unidentified sumo interaction motifs (SIMs) in WW domain-containing transcription factor (TAZ) and Yes-associated protein (YAP) to regulate protein activity and TAZ/YAP polyubiquitination in a Hippo-independent manner. Additionally, EGF therapy or KRAS mutation stabilizes YAP/TAZ expression and OTUB2 sumoylation [29]. Accordingly, via these mechanisms, OTUB2 facilitates the metastasis of MCF10A-RAS breast cancer cells. However, only four of the TMA cases in this study had metastasis, not because gastric cancer metastasis is rare [30], but because many patients with M1 stage gastric cancer opt for neoadjuvant therapy first and then determine whether to operate. Therefore, while the data do not support a positive impact of OTUB2 on gastric cancer metastasis, we cannot rule it out entirely. Instead, we speculate that the metastasis of multiple cancers induced by KRT80 is also attributable to OTUB2’s firmly support [18–20]. Following that, we will focus on the association between OTUB2 and metastasis in gastric cancer through knockout mice developing metastasis model.

What’s more, given that Lys-63 linkage is involved in DNA repair, signal transduction, and protein activity regulation [23], we will also further investigate the phenotypes and mechanisms of OTUB2 and Lys-63-related functions. We believe that OTUB2 will become a novel marker for gastric cancer.

In conclusion, we discovered that OTUB2 and KRT80 were both upregulated concurrently in GC tissues and were linked with a bleak patient prognosis. This is the first time, we demonstrate that OTUB2 functions as a brand-new catalyst of GC by deubiquitinating KRT80. Besides that, OTUB2 activates the Akt pathway, suggesting that it may be a novel therapeutic and prognostic target in GC.

## Materials and methods

### Quantitative real-time PCR (qRT-PCR) and PCR

All RNA from human tissues was extracted using the TRIzol reagent, and 1 μg of RNA was reverse transcribed into complementary DNA using the PrimeScript RT Reagent Kit (Beyotime, Shanghai, China). A quantitative real-time PCR was performed using the SYBR Green Mix (Beyotime, Shanghai, China) on an ABI Prism 7900 Sequence Detection System (ThermoFisher, CA, USA). By using the formula 2^−^^△△Ct^, we can quantify the expressions of OTUB2 and KRT80. Each sample was run in triplicate. The primer sequences used in this study were in (Table S1).

### Tissue specimens

From 2012 to 2016, GC tissues and paired normal gastric tissues were collected and randomly used in this analysis at the Peking Union Medical College Hospital. Prior to surgery, no patients underwent chemotherapy, radiotherapy, or other neoadjuvant therapies. The Ethics Committee of Peking Union Medical College Hospital (PUMCH) approved this research, and informed consent was obtained from all patients enrolled in the report.

### Stable cell line construction and cell culture

The HGC27, MKN45, NCI-N87, AGS cell lines of human gastric cancer, and HEK293T cell line were bought from the Cell Resource CAMS and PUMC (Beijing, China). The HEK293T cell line was cultured in Dulbecco’s Modified Eagle Medium, and the other cell lines in RPMI 1640 Medium, supplemented with 1% streptomycin/penicillin and 10% fetal bovine serum (Gibco, CA, USA). All cells were cultured at 37 °C in a humidified atmosphere containing 5% CO_2_.

Lentiviral preparations were developed by transfecting HEK293T cells transiently with pMD2.G and psPAX2 using Lipofectamine 2000 (ThermoFisher, MA, USA) in accordance with the manufacturer’s protocol. As controls, we used the negative control and an empty vector (nc). The sequences of shOTUB2 and shKRT80 were in (Table S1). Site-directed mutagenesis was used to generate the OTUB2-C51S mutant, which was verified by DNA sequencing.

### Western blotting

Both samples and cultured cells were lysed with RIPA lysis buffer (Beyotime, Shanghai, China) supplemented with 1% of the mixture of protease inhibitor (Sigma, MO, USA). Proteins were isolated and transferred to PVDF membranes (Millipore) in equivalent amounts using 10% SDS-PAGE.

As loading controls, antibodies against GAPDH (60004-1-Ig, Proteintech) were used. Antibodies used were: OTUB2 (PA5-99680, ThermoFisher), KRT80 (16835-1-AP, Proteintech), phospho-Akt (Thr308) (#9275, CST), Akt (10176-2-AP, Proteintech), Phospho-Akt (Ser473) (66444-1-Ig, Proteintech), mTOR (66888-1-Ig, Proteintech), phospho-mTOR (Ser2448) (ab109268, abcam), P70 S6K (14485-1-AP, Proteintech), Phospho-p70 S6(Thr389) (#9205, CST), Myc-tag (9e1, Proteintech), Flag-tag (20543-1-AP, Proteintech), His-tag (66005-1-Ig, Proteintech), and Ki67 (PA5-114437, ThermoFisher).

### Tissue microarray (TMA) construction with multiple immunofluorescence (IF) staining and immunohistochemistry (IHC)

TMA construction were performed as described previously with refs. [21, 31]. The TMA glass slides were cleaned with PBS and fixed in 4% paraformaldehyde for 10 min. After 1 h of blocking using 5% BSA (Amresco, OH, USA), the slides were incubated with anti-OTUB2, anti-KRT80, and anti-Ki67 (1:100), accompanied by goat anti-rabbit-Cy5-conjugated IgG (1:100, ZSGB-BIO) (1:100, Abcam) or goat anti-mouse-TRITC-conjugated IgG (1:100, ZSGB-BIO). Following washing, the slides were examined using an automated quantitative pathology imaging system (PerkinElmer, USA). The IF score was determined using the extent and intensity of staining (extent: 0 = no staining, 1 = 0–10%, 2 = 10–50%, and 3 = 50–100%; and intensity: 0 = negative, 1 = weak, 2 = moderate, 3 = solid). The total score was determined as the multiplication of the intensity and extent scores. 0–3 were deemed to be negative expressions. (low); 4–5 were deemed to be a weak expression (low); and 6–12 were considered to be a strong expression (high). The IF score was determined separately by two pathologists who were blinded to the patient characteristics.

### In vitro assays

The proliferation of cells was determined by counting the clone number of cells and their viability with the assistance of the CCK-8 kit (Cell Counting Kit-8, Beyotime). In 96-well plates, 2000 cells were seeded per well. At 24, 48, 72, and 96 h, the absorbance was measured by means of a spectrophotometer set to 450 nm. Colony formation was initiated by seeding three thousand cells per well into six-well plates. After 2 weeks, fixed cell clones were stained with crystal violet.

### In vivo assays

Subcutaneous tumor-bearing experiments in mice were approved by the Institutional Animal Care and Use Committee of the Peking Union Medical College and the Chinese Academy of Medical Sciences. Animal experiments were performed in compliance with Peking Union Medical College Hospital’s Institutional Animal Care and Use Committee guidelines. To develop a tumor-bearing model, subcutaneous injections of 10^6^ cells were made into the left flanks of 5-week-old male nude mice. (VitalRiver Laboratory Animal Co., Ltd., Beijing, China) (randomly divided into different groups with five mice in each group). Every 4 days, the size of the tumor was determined using Vernier calipers. The volume of the tumor was determined by way of the volume formula = width^2^ × length × 0.5. All of the experimental mice have been euthanized after 4 weeks and tumors were dissected and fixed in formalin.

### Co-immunoprecipitation (IP) assays and His pull-down assay

As previously mentioned co-IP and His pull-down assays were performed [32]. Cycloheximide (CHX, 20 μg/mL, MedChemExpress) and/or MG132 (20 μM, S2619, Selleck) were added to dishes at the specified concentrations for the indicated periods, accomplished by western blotting.

### Statistical analysis

To equate quantitative variables between groups, the Student’s *t*-test was used. ANOVA was used to compare several groups. The Chi-square test was used to analyse the correlation between the expression of OTUB2 protein and clinicopathological characteristics of GC patients. The survival outcome of patients was determined using the log-rank test and the Kaplan–Meier process. *P* < 0.05 was determined to be statistically significant.

### Ethical statement

The study involving the usage of patients’ tissues was performed in accordance with the Declaration of Helsinki and was approved by the Ethics Committee of Peking Union Medical College Hospital (approval No. 001933, date: 2019.10.22). All of the patients were given and accepted an informed consent form prior to their enrollment. The animal experiment protocol complied with the Basel Declaration and was also approved by the Ethics Committees of Peking Union Medical College Hospital (approval No. 001933, date: 2019.10.22).

### Supplementary information

Supplementary legends

Figure S1

Table S1

## Data Availability

All data and models generated and used during the study are available from the corresponding author by request.
