# Correction: OTUB2 regulates KRT80 stability via deubiquitination and promotes tumour proliferation in gastric cancer

**DOI:** 10.1038/s41420-022-01024-2

**Published:** 2022-04-19

**Authors:** Siwen Ouyang, Ziyang Zeng, Zhen Liu, Zimu Zhang, Juan Sun, Xianze Wang, Mingwei Ma, Xin Ye, Jianchun Yu, Weiming Kang

**Affiliations:** 1 Chinese Academy of Medical Sciences and Peking Union Medical College, Beijing, China; 2 Department of General Surgery, Peking Union Medical College Hospital, Beijing, China

**Keywords:** Gastric cancer, Ubiquitylation

Correction to: *Cell Death Discovery* 10.1038/s41420-022-00839-3, published online 02 February 2022

The original version of this article unfortunately contained mistakes in Table 1. In the part of “Tumor T stage”, the line “4” showed “5 13”. But in fact it should be “13 5”. It does not affect the *P* value and the conclusion. The corrected Table 1 can be found below. The authors apologize for the error. The original article has been corrected.

**Table 1:** Association between clinicopathological parameters and OTUB2 expression (*n* = 90).

	OTUB2 expression		*P* value
	Low(*n* = 49)	High(*n* = 41)	
Gender			
Male	37	33	0.572
Female	12	8	
Age, years			
<60	15	14	0.721
≥60	34	27	
Tumour location			
Fundus	6	8	0.683
Antrum	27	20	
Lesser curvature	13	9	
Greater curvature	3	4	
Tumour T stage			
1	4	0	0.001*
2	18	9	
3	14	27	
4	13	5	
Tumour N stage			
0	14	9	0.276
1	8	8	
2	10	15	
3	17	9	
Tumour M stage			
0	48	38	0.226
1	1	3	
AJCC stage			
I	5	2	0.006*
II	23	7	
III	20	29	
IV	1	3	
Differentiation			
Well	16	6	0.010*
Moderate	30	24	
Poor	3	11	

*P* value is based on Pearson’s chi-square test.

*P < 0.05 indicates a significant association among the variables.

