# Correction: OTUB2 regulates KRT80 stability via deubiquitination and promotes tumour proliferation in gastric cancer

**DOI:** 10.1038/s41420-023-01751-0

**Published:** 2024-01-08

**Authors:** Siwen Ouyang, Ziyang Zeng, Zhen Liu, Zimu Zhang, Juan Sun, Xianze Wang, Mingwei Ma, Xin Ye, Jianchun Yu, Weiming Kang

**Affiliations:** 1 Chinese Academy of Medical Sciences and Peking Union Medical College, Beijing, China; 2 Department of General Surgery, Peking Union Medical College Hospital, Beijing, China

**Keywords:** Gastric cancer, Ubiquitylation

Correction to: *Cell Death Discovery* 10.1038/s41420-022-00839-3, published online 02 February 2022

In Figure 7a, the first diagram in the second column and the fifth diagram in the second column are repeated. In the reviewer’s comments, we were asked to re-upload the images after enlarging their resolution. The authors apologise for this mistake, as we were careless in the revised article and did not place the correctly enlarged image in the position of the first image in the second column. The corrected Figure 7 can be found below
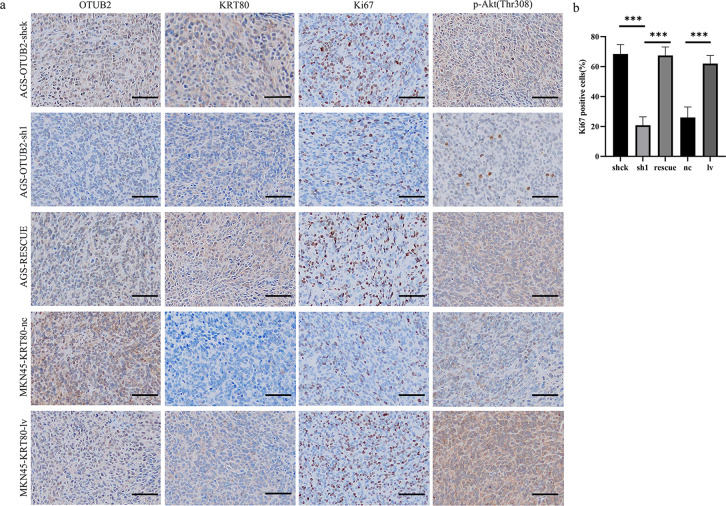


The original article has been corrected.

