# Supplementary material for: OTUB2 regulates KRT80 stability via deubiquitination and promotes tumour proliferation in gastric cancer
Source: Cell Death Discov. 2022 Feb 2;8:45. doi: 10.1038/s41420-022-00839-3 (PMC8810928; doi:10.1038/s41420-022-00839-3)
Supplement: Supplementary file 1 — Supplementary legends [file 41420_2022_839_MOESM1_ESM.docx]

**Additional file 1: Figure S1.** The mRNA levels of OTUB2 and KRT80 of the OTUB2-knockdown AGS cells. OTUB2 and KRT80 mRNA levels were determined relative to β-actin mRNA levels and then normalized to shck quantitative RT-PCR values. ****P < 0.0001; ns, not significant (two-paired Student’s t test). Data represent at least three independent experiments.

**Additional file 2: Table S1.** The primer sequences used in this study.
