# Supplementary figures and images for: OTUB2 regulates KRT80 stability via deubiquitination and promotes tumour proliferation in gastric cancer
Source: Cell Death Discov. 2022 Feb 2;8:45. doi: 10.1038/s41420-022-00839-3 (PMC8810928; doi:10.1038/s41420-022-00839-3)

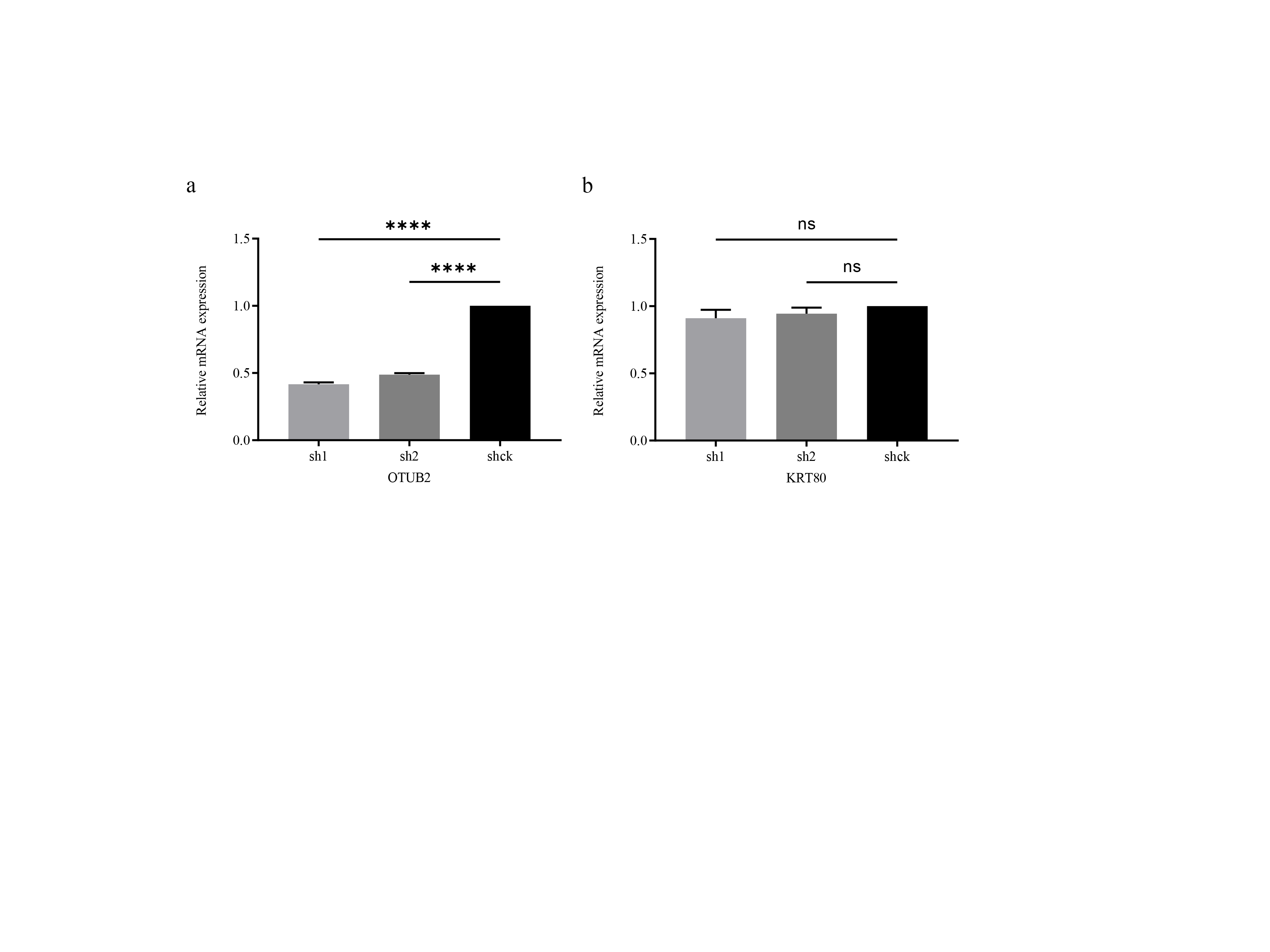

Supplement: Supplementary file 2 — Figure S1 [file 41420_2022_839_MOESM2_ESM.tif]
