# Supplementary material for: OTUB2 regulates KRT80 stability via deubiquitination and promotes tumour proliferation in gastric cancer
Source: Cell Death Discov. 2022 Feb 2;8:45. doi: 10.1038/s41420-022-00839-3 (PMC8810928; doi:10.1038/s41420-022-00839-3)
Supplement: Supplementary file 3 — Table S1 [file 41420_2022_839_MOESM3_ESM.docx]

**KRT80 primer sequences and KRT80 shRNA sequences:**

KRT80 forward: ATGGCCTGCCGCTCCTGCGTGGTT

KRT80 reverse: TTACTCTGAGACCTCCGACTCCT

shRNA1 forward: GCACTATCTCCAAGGTGACTGTGAA

shRNA1 reverse: TTCACAGTCACCTTGGAGATAGTGC

shRNA2 forward: GGATGCAGAGTGTCTTCATCG

shRNA2 reverse: CGATGAAGACACTCTGCATCC

**OTUB2 primer sequences and OTUB2 shRNA sequences:**

OTUB2 forward: TGCACTCACGAAGTAGAGCC

OTUB2 reverse: TGAAGAGCCGGAATGTTCCAT

shRNA1 forward: ACCACATCGTGCAGTTCCT

shRNA1 reverse: TGTGACATTCTATCCATTC

shRNA2 forward: GTGAGTACGTGGACATCAA

shRNA2 reverse: CCAACTACCTGAACGATGA

**β-actin primer sequences:**

β-actin forward: GTCATTCCAAATATGAGATGCGT

β-actin reverse: GCATTACATAATTTACACGAAAGCA
